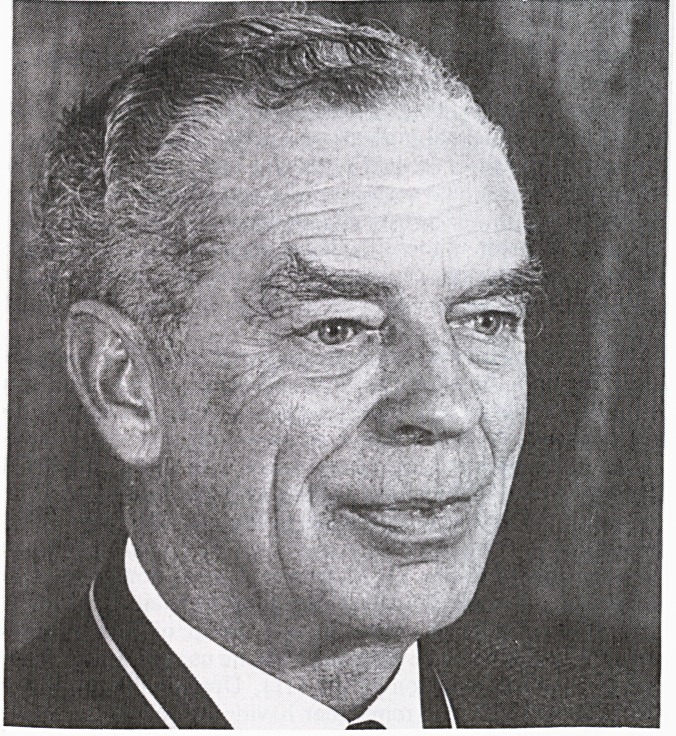# Dr W. H. Hylton

**Published:** 1989-08

**Authors:** 


### Obituary

### **William H. Hylton** BSc (Engineering) Leeds, MRCS (Eng), LRCP (Lond), FRCGP

Dr William H. Hylton, formerly a general practitioner in Clevedon died suddenly on March 20th at the age of 83.

William Hawkins Hylton—Bill, was born in Leeds in 1906. He was educated at Bridlington College. His father ran a building firm but died tragically of acute appendicitis when his son was aged 14. Bill intending to pursue the same career entered Leeds University and took a degree in engineering. Before he had completed it however the family firm had ceased to operate. Bill started another building firm of his own and when this failed he decided to start a new career. In 1933 he entered Leeds Medical School and qualified in 1939 at the age of 33. The author remembers him as a student in a year or two ahead, a striking figure, mature amongst a lot of youths, debonair and immaculate and able to indulge his hobby of motor racing in his Fraser Nash. On qualifying he joined the Territorial RAMC and shortly after the outbreak of War was serving in France. With the collapse of France after the German invasion he was involved in the retreat from Dunkirk and was mentioned in dispatches. Later he served in India and Burma where he became a DADMS with the rank of Lieutenant Colonel. It was in India that he met his wife Lorna, also a Leeds medical graduate, who was serving in the Indian Medical Service at Ahmednaghar near Poona where they were married in 1944. Later Bill was posted to Burma and was at Kohima and Imphal in the army commanded by General Slim at the 'turn of the tide'. On demobilisation, they bought a practice in Clevedon where they practiced together until Bill retired in 1977. In 1971 when Clevedon Health Centre was built they joined with another practice and became a large partnership.

Bill was a founder member of the Royal College of General Practitioners. He was Provost of the Severnside Faculty and was responsible for running symposia in the area. He was a member of the Council of the R.C.G.P. and National Chairman of the Committee for Vocational Training. He was a Fellow of the Royal Society of Medicine and a sometime chairman of the Bath, Bristol and Somerset branch of the BMA. His natural kindliness and unfailing courtesy more than offset the direct no-nonsense approach native to a Yorkshireman. He shared with Lorna the hobbies of gardening and of birdwatching, travelling all over the world with camera and binoculars in pursuit of rare species in faraway places. He is survived by Lorna and by five children, one of whom is a doctor and nine grandchildren.

## Figures and Tables

**Figure f1:**